# *NANOG* Reporter Cell Lines Generated by Gene Targeting in Human Embryonic Stem Cells

**DOI:** 10.1371/journal.pone.0012533

**Published:** 2010-09-02

**Authors:** Yvonne Fischer, Elvira Ganic, Jacqueline Ameri, Xiaojie Xian, Martina Johannesson, Henrik Semb

**Affiliations:** 1 Stem Cell Center, University of Lund, Lund, Sweden; 2 Biotech Research and Innovation Center, University of Copenhagen, Copenhagen, Denmark; 3 Department of Stem Cell Biology, Hagedorn Research Institute, Gentofte, Denmark; Wellcome Trust Centre for Stem Cell Research, United Kingdom

## Abstract

**Background:**

Pluripotency and self-renewal of human embryonic stem cells (hESCs) is mediated by a complex interplay between extra- and intracellular signaling pathways, which regulate the expression of pluripotency-specific transcription factors. The homeodomain transcription factor NANOG plays a central role in maintaining hESC pluripotency, but the precise role and regulation of NANOG are not well defined.

**Methodology/Principal Findings:**

To facilitate the study of NANOG expression and regulation in viable hESC cultures, we generated fluorescent NANOG reporter cell lines by gene targeting in hESCs. In these reporter lines, the fluorescent reporter gene was co-expressed with endogenous NANOG and responded to experimental induction or repression of the NANOG promoter with appropriate changes in expression levels. Furthermore, NANOG reporter lines facilitated the separation of hESC populations based on NANOG expression levels and their subsequent characterization. Gene expression arrays on isolated hESC subpopulations revealed genes with differential expression in NANOG^high^ and NANOG^low^ hESCs, providing candidates for NANOG downstream targets hESCs.

**Conclusion/Significance:**

The newly derived NANOG reporter hESC lines present novel tools to visualize NANOG expression in viable hESCs. In future applications, these reporter lines can be used to elucidate the function and regulation of NANOG in pluripotent hESCs.

## Introduction

Embryonic stem cells have the unique capability to replicate indefinitely while maintaining pluripotency, i.e. the potential to develop into all cell types of the adult organism. In human embryonic stem cells (hESCs), external ligands like Activin A/TGFβ/Nodal, FGF2 and Insulin/IGF cooperate to activate downstream transcription factors, thereby creating a complex signaling network that ultimately maintains the pluripotent state. One major component of the pluripotency signaling network is the homeodomain transcription factor NANOG [1], [2], which together with OCT4 and SOX2 constitutes the core transcription factor network in hESCs [3]. Experimental knockdown of NANOG expression leads to hESC differentiation to embryonic and/or extraembryonic lineages, depending on the experimental conditions and on cell line-intrinsic determinants [4], [5], [6]. Conversely, overexpression of NANOG in hESCs promotes self-renewal in the absence of feeders [7] and eliminates the requirement for Activin A in feeder-free systems [6], [8]. Moreover, NANOG expression is required to establish full pluripotency during reprogramming of fibroblasts to induced pluripotent stem (iPS) cells, as well as for the formation and stabilization of pluripotent epiblast and germ cells *in vivo* [9], [10], [11]. Thus, it seems that NANOG expression serves both as a determinant and an indicator for bona fide pluripotency, albeit the exact role of NANOG in establishing and maintaining pluripotency remains to be determined.

Reporter cell lines, in which a reporter gene is expressed from a specific endogenous promoter, are valuable tools to study gene regulation and function in real-time in living cells, which cannot be achieved by conventional biochemical and immunological methods. Reporter cell lines have been successfully applied in embryonic stem cell research to identify inducers and repressors of specific promoters (e.g. in high throughput screens with chemical or RNAi libraries) and to separate subpopulations of differentiated cells [12], [13], [14], [15], [16], [17]. Thus, Nanog reporter lines were created and applied to screen for signaling pathways inducing mouse embryonic stem cell (mESC) differentiation [18], to delineate the role of Nanog in pluripotency of mESCs and during embryogenesis [9], [19], [20], and to monitor iPS cell generation during reprogramming experiments [10], [21]. However, pluripotency and differentiation is regulated through different pathways in murine and human cells, which is reflected by different marker expression and response to signaling molecules of mESCs versus hESCs (reviewed in [22]). This restricts the application of principles from mESC biology to hESCs.

To enable the study of NANOG expression and NANOG-mediated pluripotency in hESCs, we derived NANOG reporter cell lines by gene targeting in hESCs. We chose to pursue a gene targeting strategy rather than random transgenic integration of the reporter construct to avoid uncontrollable position effects on reporter expression, and to enable the accurate expression of the reporter gene from the endogenous regulatory sequences of the *NANOG* locus. These novel NANOG reporter cell lines constitute efficient tools to study the role and regulation of NANOG in human pluripotent cells.

## Materials and Methods

### Human embryonic stem cell culture and differentiation to embryoid bodies

The hESC lines HUES-1 and HUES-3 used in this study were obtained from the Howard Hughes Medical Institute (Harvard University, Cambridge, MA) and derived as previously described [23]. HESCs were grown on mitomycin C-treated murine embryonic fibroblast (MEF) feeders in medium containing KO-DMEM, 20% knockout serum replacement, 10ng/ml bFGF, 1% non-essential aminoacids, 1% Glutamax, 0,1% beta-Mercaptoethanol and 1% Penicillin/Streptomycin (all from GIBCO, Invitrogen). Cells were passaged with 0,05% trypsin/EDTA (GIBCO, Invitrogen) and re-plated at a split ratio of 1∶3 to 1∶6. For feeder-free culture, hESCs were transferred to matrigel (Becton Dickinson)-coated culture dishes and fed with mTeSR1 medium (Stem Cell Technologies). Cells were passaged with dispase at a split ratio of 1∶2 to 1∶3.

For Activin A response experiments, cells were seeded in matrigel-coated 24-well plates at a density of 100.000 cells/well in mTeSR1 medium. Activin A was added to the medium 24 hours after seeding of cells. Expression of eGFP was analyzed 48 hours after addition of Actvin A by flow cytometry.

For embryoid body (EB) differentiation, cells were plated at a density of one million cells/ml in Petri dishes and cultured with hESC culture medium without bFGF or in embryoid body medium containing 20% FBS as previously published [24]. Samples for flow cytometry and PCR were taken on days 0–28 of differentiation and analyzed as described below. For immunofluorescence staining, EBs from day 22 (NANeG3 cells) or day 10 (NANeG1 cells) were plated on matrigel-coated glass cover slips and incubated for additional 6–7 days with hESC medium or EB medium.

Karyotyping of hESC clones was performed by standard G-banding in collaboration with the Institute for Clinical Genetics at the Universities of Lund, Sweden. For each analysis, 20–25 metaphases were evaluated.

### BAC recombineering

All recombineering reagents including the recombineering-competent *Escherichia coli* (*E. coli*) strain SW102 were obtained from the Biological Resources Branch preclinical repository of the National Cancer Institute (Maryland, USA). A detailed description of materials is given on the website http://recombineering.ncifcrf.gov. The bacterial artificial chromosome (BAC) CTD-2317D19 containing the human *NANOG* locus was obtained from Invitrogen. The identity of the BACs was verified by restriction enzyme digestion and sequencing. The retrieval vector pBR322 was obtained from New England Biolabs. All recombineering experiments were performed according to protocols published previously [25], [26], [27], [28] and http://recombineering.ncifcrf.gov. The eGFP-pSV40-Neo^R^ reporter cassette was obtained by conventional restriction cloning. The rabbit beta globin intron 2 was cloned into the XhoI site of pEGFP-N1 (Clonetech). The pSV40-Neo^R^ selection cassette was PCR-amplified from pEGFP-N1 with chimeric primers containing loxP sites and recognition sites for the restriction enzymes DraIII and BsaI (see supplementary Text S1 for primer sequences). The resulting PCR product was digested and ligated into DraIII and BsaI sites of pEGFP-N1, replacing the original pSV40-Neo^R^ cassette. The eGFP-pSV40-Neo^R^ reporter cassette and the retrieval plasmid pBR322 were amplified by PCR (Accuprime Pfx Polymerase, Invitrogen) prior to recombineering. The primers used for these PCR reactions contained 50bp of homology to the respective target sequence within the BAC (see supplementary Text S1 for primer sequences). The reporter cassette was inserted 5′ to the start codon of the *NANOG* gene into the BAC. For retrieval into pBR322, recombineering target sites within the BAC lying 12.5kb upstream and 3.5kb downstream of the reporter cassette were chosen. The integrity of the finalized targeting constructs was verified by PCR, restriction analysis and sequencing.

### Gene targeting

The *NANOG* targeting vector was purified from *E.coli* (Genomed Jetstar DNA preparation kit) and linearized with I-SceI. Five hours before electroporation, medium on hESCs was changed and the Rock-inhibitor Y-27632 (Calbiochem), which increases the survival of hESCs after single-cell dissociation [29], was added at a concentration of 10µM. Exponentially growing hESCs were harvested with trypsin/EDTA, washed with phosphate buffered saline (PBS) and counted. HESCs were resuspended in 700µl of ice-cold PBS containing 40µg of the targeting vector. Between four and six million hESCs were used for each transfection. Electroporation was performed in 0.4 cm cuvettes on a Gene Pulser XCell (BioRad) with the parameters 250V, 500µFd or 800V, 10µFd. After electroporation, cells were washed once with pre-warmed medium and plated on 10cm dishes containing neomycin-resistant MEFs in the presence of Y-27632 (10µM). Two to three days after transfection, selection with Geneticin (Invitrogen) (100 µM) was started and maintained for 7–10 days. Emerging clones with undifferentiated morphology were counted and examined for eGFP expression. Clones expressing eGFP were picked and plated in 24-well plates on MEFs in the presence of Y-27632. When the clones reached sub-confluence, they were detached by trypsin/EDTA treatment and expanded. PCR to detect *NANOG* gene targeting over the 3′ flanking region was performed on genomic DNA of hESC clones using Elongase (Invitrogen) and the cycle conditions 93°C/1min, 58°C/30sec, 68°C/7min (repeat 45 times). Gene targeting of the 5′ flanking region was detected using the Failsafe PCR system (Epicentre Biotechnologies) and the cycle conditions 93°C/1min, 62°C/30sec, 70°C/16min (repeat 30 times). Primer sequences are shown in supplementary Text S1. The identity of the obtained PCR products was verified by XhoI+SpaI digestion of the 5′PCR product and HindIII digestion of the 3′PCR product.

### Immunofluorescence

HESCs growing on tissue culture dishes or on matrigel-coated glass cover slips were fixed with 4% paraformaldehyde/PBS for 15 minutes, permeabilized with 0,05% Triton-X-100/PBS for 20 minutes and pre-incubated with 5% skim milk/PBS for one hour. Primary antibodies were added in 5% skim milk/PBS at the following dilutions: NANOG (Sigma N3038) 1∶300; Oct4 (Santa Cruz sc-5279) 1∶500; Tra-1-60 (Santa Cruz sc-21705) 1∶250; Tra-1-81 (Santa Cruz sc-21706) 1∶250; SSEA-4 (Developmental studies hybridoma bank MC-813-70) 1∶200; βIII-Tubulin (Promega G7121) 1∶1000; Sox1 (Abcam ab22572) 1∶200; α-smooth muscle actin (Sigma F3777) 1∶200; α-fetoprotein (Sigma A8452) 1∶400, Albumin (Bethyl A80-129-F) 1∶200. Incubation with primary antibodies was performed over night at 4 degrees. Cells were washed three times with PBS. Secondary antibodies were added for 1h at room temperature in PBS at the following dilutions: anti-mouse-Cy3 (Jackson Laboratories 715-165-150) 1∶300–500; anti-rabbit-Alexa 488 (Molecular Probes A11008) 1∶500; anti-mouse-Alexa 647 (Molecular probes A-31571) 1∶500. Cells were washed three times with PBS and mounted in the presence of DAPI nucleic acid stain. Images were taken using an Axioplan 2 Fluorescence microscope and Axiovision software.

### Semi-quantitative and quantitative polymerase chain reaction

Total RNA was isolated from hESCs or embryoid bodies using the Genelute total RNA kit (Sigma). RNA was digested with DNAseI (Invitrogen) and reverse transcribed to cDNA using Superscript III reverse transcriptase (Invitrogen). Semi-quantitative PCR was performed with Taq-polymerase (Sigma). Primers, cycle numbers and annealing temperatures for semi-quantitative PCR are listed in supplementary Text S1. PCR products were separated on agarose gels in presence of ethidium bromide and analyzed under UV light. For quantitative real-time PCR, reactions were performed with Platinum quantitative PCR SuperMix (Invitrogen Cat.no. 11743) in the presence of SYBR green (Invitrogen). PCR cycles on a 7900HT fast real time PCR system (Applied Biosystems) were performed as follows: 50°C/2min, 95°C/2min, 95°C/15sec, 60°C/25sec, 73°C/30sec (steps 3–5 were repeated 40 times). Gene expression levels were normalized to endogenous GAPDH expression and quantified using the ΔΔCt method [30]. Primers used for quantitative real-time PCR are listed in supplementary Text S1.

### Copy-number determination by quantitative PCR

The copy number of the NANOG targeting vector in NANeG cells was determined by quantitative PCR following the guidelines for assay design, controls and evaluation given in [31]. A fragment of the NANOG proximal promoter (−758 to −858) was amplified and quantified relative to the copy number of the single-copy reference genes *GDF3* (GeneID: 9573) and *FOXJ2* (GeneID: 55810). Primers used for qPCR reactions are shown in supplementary Text S1. PCR cycles on a 7900HT fast real time PCR system (Applied Biosystems) or a Step1Plus real time PCR system (Applied Biosystems) were performed as follows: 50°C/2min, 95°C/2min, 95°C/15sec, 60°C/25sec, 73°C/30sec (steps 3–5 were repeated 40 times). Measurements on all samples were repeated in at least three qPCR experiments which were performed in triplicates. A standard curve with serial dilutions of hESC genomic DNA ranging from 0.78 to 25ng/reaction in 2-fold dilution steps was prepared by plotting mean threshold cycle (Ct) values against log-transformed gDNA concentrations. A linear trend line was fitted into each standard curve and slopes (y) and correlation coefficients (R^2^) were obtained in Microsoft Excel. Assay efficiencies were calculated using the formula E = 10^(−1/y)^. Melt curve analysis and absence of amplification products from water controls confirmed the specificity of the assays. Copy numbers of the NANOG promoter fragment (pNANOG) in test samples were normalized to reference assays (GDF3 or FOXJ2) and compared to untransfected hESCs (control) using the amplification efficiency-adjusted ΔΔCt method with the formula:
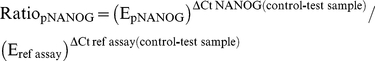

[32].

Statistical significance of different pNANOG quantities between samples was analysed with student's t-test.

### Flow cytometry and cell sorting

For quantification of eGFP expression, hESCs growing in feeder-free culture or embryoid bodies were dissociated with Trypsin/EDTA and resuspended in PBS. Expression of eGFP was measured on a FACSCalibur flow cytometer (Becton Dickinson) using CellQuest software. Ten thousand living cells were counted and analyzed for eGFP expression, using untransfected hESCs as negative controls. For cell sorting, NANeG cells were dissociated with trypsin/EDTA and resuspended in PBS containing 2% fetal bovine serum and the Rock-inhibitor Y-27632 (10µM). Cell sorting of eGFP^high^ and eGFP^low^ hESCs was performed on a DiVa flow cytometer with DiVa software (Becton Dickinson). Approximately 200.000 cells were sorted from each subpopulation and used for mRNA extraction. Re-analysis after cell sorting confirmed that the purity of the sorted populations was >95%.

### Gene expression arrays

Gene expression arrays were performed with TaqMan Human Stem Cell Pluripotency Array plates (Applied Biosystems # 4414077) according to the manufacturer's instructions. Briefly, cDNA from FACS-sorted NANeG subpopulations was combined with gene expression mastermix (Applied Biosystems #4369016) and loaded onto array plates containing gene-specific primers and probes. Gene expression was measured on a 7900HT fast real time PCR system (Applied Biosystems) with the cycle conditions 50°C/2min, 95°C/10min, 95°C/15sec, 60°C/1min (steps 3–4 were repeated 40 times). Gene expression levels were normalized to the threshold cycle (Ct) values of endogenous *GAPDH* and quantified using the ΔΔCt method [30].

## Results

### Gene targeting of the *NANOG* locus in hESCs

**Figure 1:**
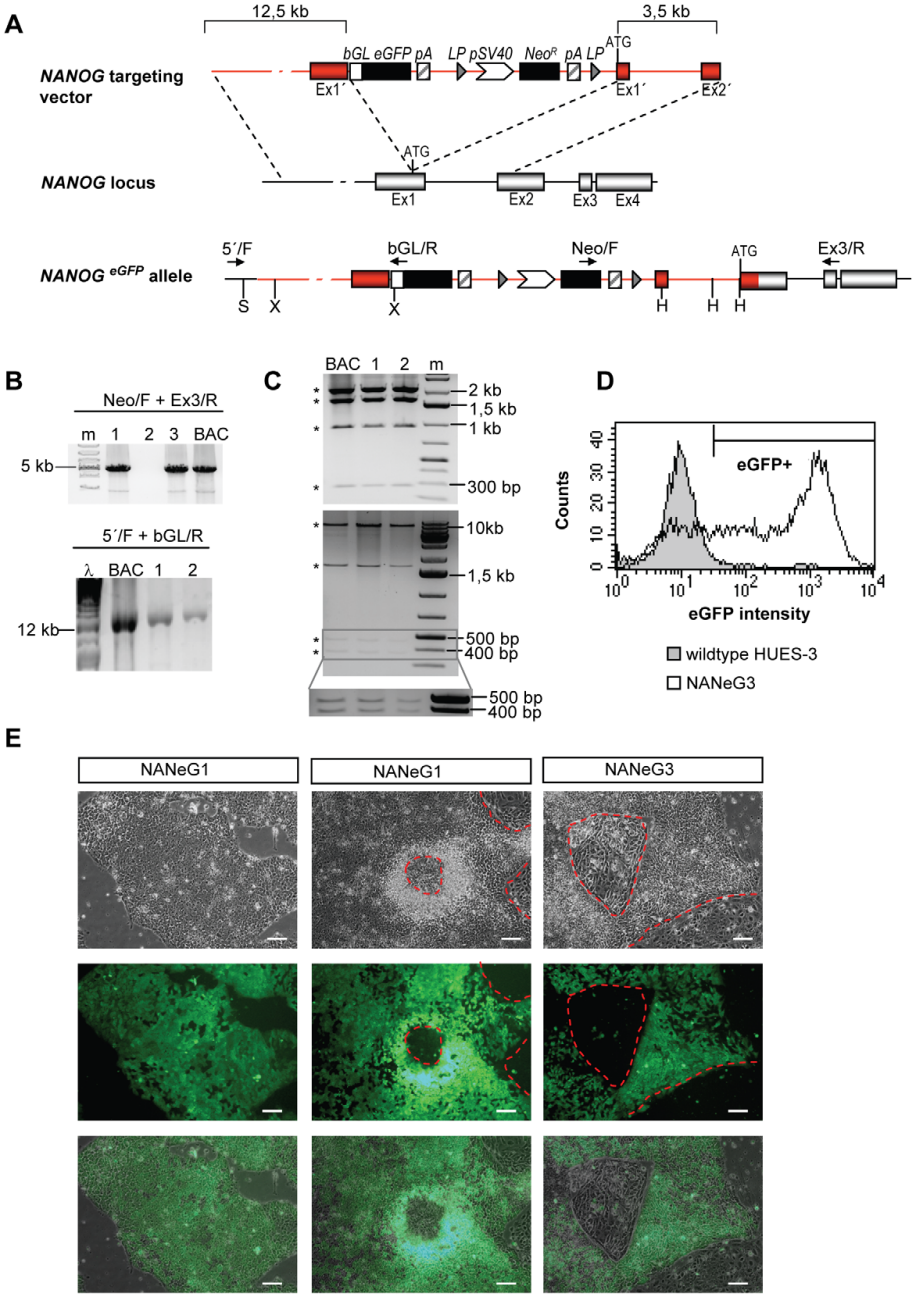
Gene targeting of *NANOG* in hESCs and expression of the eGFP reporter. The strategy for gene targeting of the *NANOG* locus is presented schematically in A. The *NANOG* targeting vector was inserted into the 5′ untranslated region of the *NANOG* gene upstream of the *NANOG* start codon (ATG), resulting in an eGFP-tagged *NANOG* allele. Primer binding sites for PCR-based screening are indicated as arrows (A, lower panel). Red coloring marks targeting vector–derived *NANOG sequences* which replace endogenous *NANOG* sequences upon gene targeting. Abbreviations: bGl, rabbit beta-globin Intron 2; eGFP, enhanced green fluorescent protein gene; LP, locus of X-over P1 (recognition site for Cre-recombinase); Neo^R^, neomycin resistance gene; pA, polyadenylation site; Ex, exon; Ex1′, Ex2′, tuncated exon 1, 2. B) PCR experiments to screen for *NANOG* gene targeting events. A representative PCR screen of 3′ targeting events is shown in B, upper panel for three individual clones (lane 1 and 3: clones positive for 3′ gene targeting; lane 2: clone negative for 3′ gene targeting) with Neo/F and Ex3/R primers (fragment size 5,1kb). The *NANOG^eGFP^* BAC containing the reporter cassette integrated into the *NANOG* locus was used as positive control (BAC). m, DNA size marker. Correct targeting of the 5′ flanking region was tested by long-range PCR as shown in B, lower panel. PCR products of 13.3 kb were obtained with 5′/F and bGL/R primers with genomic DNA from NANeG1 and NANeG3 clones (lane 1, 2) and with the *NANOG^eGFP^* BAC template as positive control. C) The identity of the PCR products was verified by restriction enzyme digestion of the 3′PCR product with Hind III (upper panel) and SpeI/XhoI digestion of the 5′PCR product (lower panel). Expected fragments for 3′PCR products were 269bp, 965bp, 1.7kb and 2.2kb (marked by stars). Expected fragments for 5′PCR products were 374bp, 470bp, 1.7kb and 10.9kb (marked by stars). The 374bp and 470bp fragments are shown with higher magnification and contrast in the insert in C lower panel. Lanes contain PCR-products obtained with *NANOG^eGFP^* BAC (BAC), NANeG1 (1), NANeG3 (2), DNA size marker (m). XhoI (X) SpeI (S) and HindIII (H) sites within the gene targeted *NANOG* locus are indicated in A, lower panel. D) Histogram of eGFP expression levels in NANeG3 cells measured by flow cytometry. Wild-type HUES-3 cells were used as negative control. E) Brightfield (upper panel) and corresponding epifluorescence (middle) and merged (lower panel) images of NANeG1 and NANeG3 in a feeder-free culture system. Dashed lines indicate areas of spontaneous differentiation with loss of eGFP expression. Scale bar: 100µm.

The *NANOG* targeting vector used for this study (Fig. 1A) was created by bacterial artificial chromosome (BAC) recombineering (i.e. BAC engineering by homologous recombination) [33]. BAC recombineering is a fast and efficient method to create gene targeting vectors containing >10kb of homologous DNA, which is required for optimal gene targeting efficiency [34]. A BAC containing the human *NANOG* gene plus >10 kb of flanking sequences was identified using the genome browser at http://genome.ucsc.edu. A reporter cassette, consisting of an enhanced green fluorescent protein (*eGFP*) gene and a neomycin resistance gene was inserted into the BAC immediately upstream of the *NANOG* start codon. The finalized targeting vector, containing homology arms of 12.5kb and 3.5kb, was retrieved into a bacterial plasmid through a second round of recombineering.

The hESC lines HUES-1 and HUES-3 were transfected with the p*NANOG* ^eGFP^ vector by electroporation. Clones arising from transfections were scored for eGFP expression and eGFP positive clones were isolated and expanded. Gene targeting of the *NANOG* locus was assessed by PCR amplification of the 3′ region flanking the reporter cassette. A PCR product of 5.1kb size was amplified from genomic DNA of *NANOG* gene targeted clones as shown in Figure 1B. The identity of the PCR product was verified by restriction enzyme digestion (Fig. 1C) and sequencing (data not shown). The results of gene targeting experiments are summarized in Table 1. Clones with a targeted *NANOG* allele (designated NANeG) were obtained from both HUES cell lines. The relative targeting frequency was significantly higher in HUES-3 (11.4% of drug-resistant clones) than in HUES-1 (0.6% of drug-resistant clones). To test if additional copies of the targeting vector have been incorporated in the genome of NANeG cells by random insertion, a quantitative PCR assays to detect a fragment of the NANOG proximal promoter (pNANOG) in genomic DNA of hESCs was established. In addition, quantitative PCR assays for two single-copy reference genes (*GDF3* and *FOXJ2*) were established to measure relative quantities of pNANOG in untransfected hESCs and in NANeG clones obtained from transfections with the *NANOG* targeting vector. Preparation of standard curves with hESC genomic DNA revealed amplification efficiencies between 90 and 100% and R^2^ values of 0.99 for all assays (Fig. S3A), showing that the assays fulfilled the quality requirements for copy number determination on genomic DNA [31]. Relative quantities of pNANOG were determined for four NANeG clones (NANeG1, 3, 11 and 31) and three clones which showed eGFP fluorescence but were negative for gene targeting (Transgenic clones 12, 13 and 32) (Fig. S3B). As expected, all transgenic clones contained significantly increased quantities of pNANOG sequences in genomic DNA samples (p<0.01 for pNANOG vs. *GDF3* and p<0.05 for pNANOG vs. *FOXJ2*). NANeG clones 1, 3 and 31 showed no significant increase of pNANOG sequences above control hESCs (p>0.1) in both assays, whereas NANeG11 contained significantly elevated pNANOG levels in genomic DNA samples (p<0.01 in both assays). These results indicate that NANeG1, 3 and 31 contain a single copy of the NANOG targeting vector integrated into the endogenous NANOG locus by gene targeting, whereas NANeG11 contains additional copies of the targeting vector.

**Table 1:** Gene targeting of the *NANOG* locus in hESCs.

	HUES-1	HUES-3
Transfected cells	19 Million	9 Million
G418^R^ clones	637	167
G418^R^GFP^+^ clones	33	40
Targeted clones	4	19
Absolute targeting efficiency^a^	2,1E-7	2,1E-6
Relative targeting efficiency^b^	0,6%	11,4%

a Targeted clones divided by number of transfected cells.

b Percentage of targeted clones among G418^R^ clones.

Karyotype analysis on a subset of gene targeted clones was performed in passage 6–7. Both cell lines gave rise to clones with normal karyotype, but chromosomal aberrations (frequently involving gain of chromosomes 12, 17 or 20) were also observed in a subset of clones derived from both cell lines. The clones NANeG1 (derived from HUES-1) and NANeG3 (derived from HUES-3) showed a normal diploid karyotype and were chosen for further analysis. Correct gene targeting of the 5′region flanking the reporter cassette was confirmed by long-range PCR (Fig. 1B) and the identity of the 13.3kb PCR fragment was further verified by restriction enzyme digestion (Fig. 1C).

Both NANeG lines expressed the hESC markers OCT4, SSEA-4, TRA-1-60 and TRA-1-81 (Fig. S1A and S2A). Pluripotency of both lines was tested by embryoid body differentiation, where they gave rise to cell types representative of the three embryonic germ layers (Fig. S1B, C and S2B, C).

### Characterization of reporter gene expression following *NANOG* gene targeting

NANeG cells grew in compact colonies in a feeder-free culture system (Fig. 1D) and expressed eGFP in most cells within the undifferentiated colonies. In areas of spontaneous differentiation, eGFP expression was downregulated. Similar to Nanog-eGFP mESCs [9], [20], NANeG cells expressed the eGFP reporter in a graded fashion, with subpopulations of cells expressing high, low or no eGFP at a given timepoint (Fig. 1C). Thereby, mean eGFP intensities were of up to 100-fold higher than auto-fluorescence levels measured in the negative control. The relative distribution eGFP high, low and negative cells varied between cell lines, passages and culture conditions (data not shown), most likely due to variable levels of spontaneous differentiation within the cultures.

**Figure 2:**
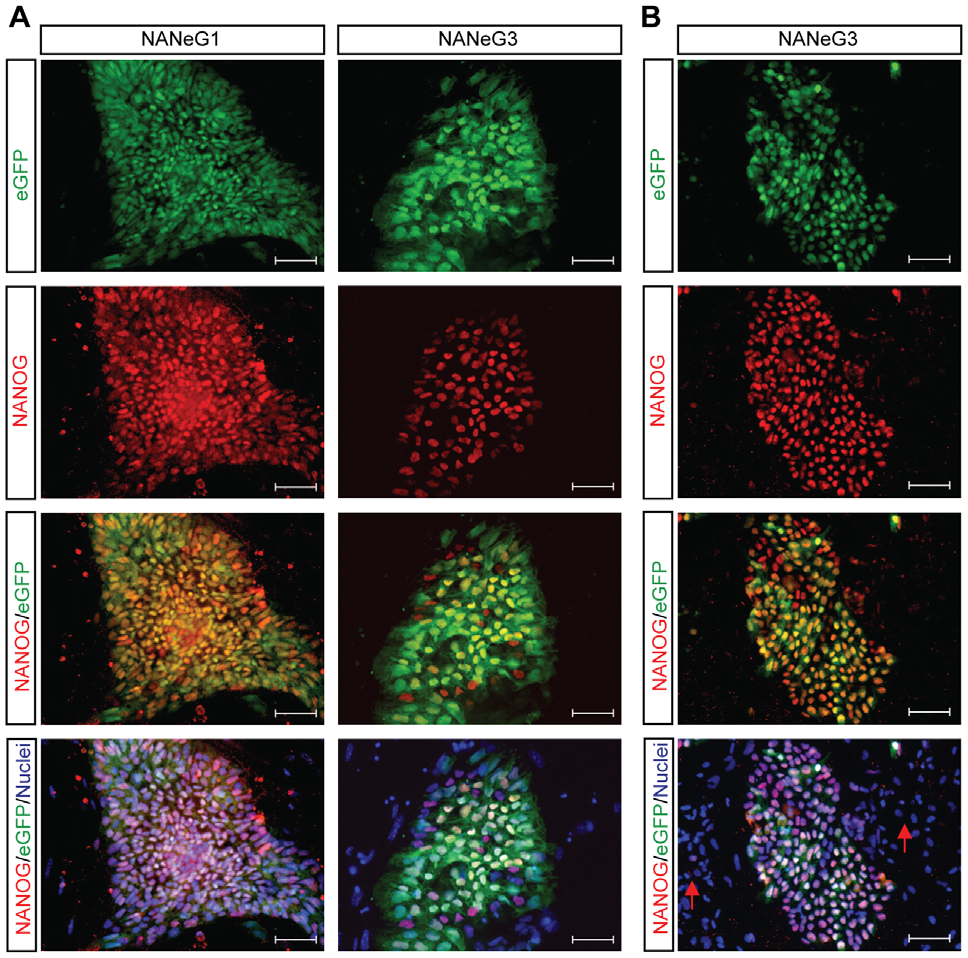
Co-expression of NANOG and eGFP in NANeG lines. Immunofluorescence staining of endogenous NANOG expression and co-localization with eGFP in NANeG lines growing on murine feeders (A) or in feeder-free culture (B). Arrows indicate areas of spontaneous differentiation with concomitant NANOG and eGFP downregulation. Scale bar: 100µm.

Immunostaining for NANOG revealed extensive co-expression of NANOG and eGFP in undifferentiated NANeG cells growing on murine fibroblast feeders (Fig. 2A) and in feeder-free culture (Fig. 2B). In areas of spontaneous differentiation (arrows in Fig. 2B), both NANOG and eGFP expression were downregulated. In both NANeG lines, a subset of cells stained positive for NANOG but express low levels or no eGFP. The same observation was made in sub-clones of NANeG3 created by single-cell deposition of eGFP-positive cells in 96-well plates (data not shown), indicating that this discrepancy was not due to the presence of contaminating wild-type hESCs within the NANeG lines.

**Figure 3:**
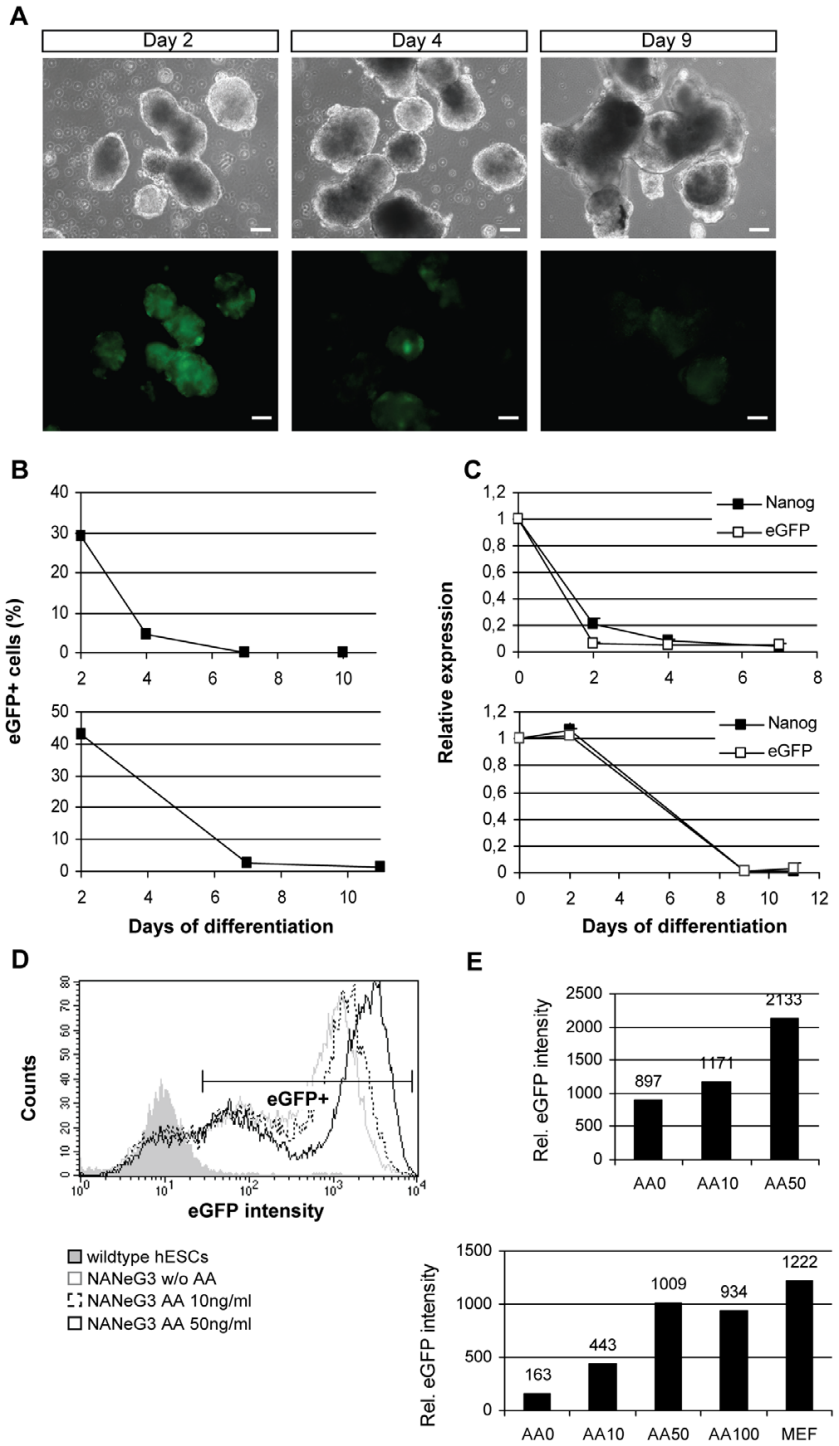
Modulation of reporter expression in NANeG lines. A) Brightfield and corresponding epifluorescence images of NANeG1 undergoing differentiation as embryoid bodies are shown for day 2, 4 and 9 of differentiation. Scale bar: 100µm. Downregulation of eGFP expression during embryoid body differentiation was quantified by flow cytometry (B) and quantitative real-time PCR (C) in NANeG1 (upper panel) and NANeG3 (lower panel). D, E) NANeG cells growing in feeder-free culture were exposed to 0, 10, 50 or 100ng/ml Activin A (AA). Activin A induced eGFP expression in a dose-dependent manner, as shown by flow cytometry (D). Mean fluorescence intensities of the eGFP+ population (indicated in D) in NANeG3 (upper panel) and NANeG1 (lower panel) are shown in (E). For comparison, NANeG1 cells were grown on murine embryonic fibroblast feeders (MEF) without addition of Activin A.

To test the adequate responsiveness of the eGFP reporter, NANeG cells were exposed to culture conditions that modulate NANOG expression in hESCs. Differentiation to embryoid bodies led to the rapid downregulation of eGFP (Fig. 3A, B) in both cell lines. Thereby, downregulation of eGFP expression followed downregulation of endogenous *NANOG* expression in response to hESC differentiation (Fig. 3C). Conversely, NANOG expression in hESCs can be activated by Activin A [6], [8]. We therefore tested the effect of Activin A on eGFP expression in NANeG cells. As shown in Figure 3D and E, Activin A caused a dose-dependent increase of eGFP expression up to a concentration of 50ng/ml, confirming the responsiveness of the eGFP reporter to exogenous signals that activate NANOG expression in hESCs.

### Gene expression profiling of hESC subpopulations with different *NANOG* expression levels

Reporter cell lines enable the fractioning of hESC populations based on endogenous gene expression. In embryonic stem cells, subpopulations with varying levels of Nanog expression co-exist and have been isolated and characterized in the murine system [9], [20], but not from hESCs due to the lack of suitable reporter lines. To study the heterogeneity of hESCs as a function of NANOG expression, NANeG cultures were fractioned into eGFP^high^ and eGFP^low^ subpopulations by flow cytometry (Fig. S4). NANOG expression in the eGFP^low^ fraction was reduced to approximately 30 percent of expression levels in the eGFP^high^ fraction (Table 2). Since NANOG is an important mediator of hESC self-renewal, we analyzed the expression profile of 96 genes involved in hESC self-renewal or differentiation in the isolated hESC fractions. Gene expression patterns of stem cell markers and early differentiation markers in NANeG lines largely resembled the expression pattern detected in parental HUES lines [35]. Furthermore, several markers for neural (*TH*, *SYP*, *NEUROD*), hepatic (*TAT*), pancreatic (GCG, *INS*, *PDX1*, *PTF1a*), blood (*HBB*, *HBZ*), and muscle lineages (*MYF5*, *MYOD*) were low or absent in NANeG cultures.

**Table 2:** Genes differentially expressed (≥2-fold up/down-regulated in both cell lines) in NANOG^high^ and NANOG^low^ hESCs.

		Expression NANOG^low^/NANOG^high^		
Gene symbol	Cellular process^a^	NANeG1	NANeG3	Assay^b^
NODAL^c^	TGFβ signaling pathway	0,06	0,16	T, Q
LEFTY1^d^	TGFβ signaling pathway	0,11	0,14	T
CDH1^d^	Cell adhesion	0,10	0,20	Q
TDGF1^d^	TGFβ signaling pathway	0,09	0,27	T, Q
POU5F1^d^	Transcription factor	0,10	0,30	Q
GFAP	Intermediate filament	0,18	0,23	T
GAL	Messenger neuropeptide	0,14	0,27	T
UTF1	Transcription factor	0,15	0,34	T
CD9	Cell adhesion	0,19	0,31	T
IFITM1	Transmembrane protein	0,16	0,39	T
T^c^	Transcription factor	0,18	0,43	T, Q
GDF3^c^	TGFβ signaling pathway	0,20	0,43	T, Q
NANOG^d^	Transcription factor	0,36	0,29	T, Q
PODXL^c^	Cell adhesion	0,30	0,37	T
GABRB3	GABA receptor	0,39	0,29	T
EOMES^d^	Transcription factor	0,26	0,43	T, Q
FLT1	Receptor tyrosine kinase	0,31	0,39	T
MIXL1	Transcription factor	0,30	0,40	Q
GRB7	Growth factor receptor adapter	0,30	0,49	T
ZFP42^c^	Transcription factor	0,30	0,50	T, Q
DNMT3B	DNA methylation	0,45	0,47	T
COL1A1	Extracellular matrix	3,12	2,37	T
LAMA1	Extracellular matrix	3,69	2,33	T
ACTC1	Cytoskeleton	3,03	3,04	T
COMMD3^d^	Protein binding	3,82	2,88	T
FN1	Extracellular matrix	3,96	3,35	T
CDH5	Cell adhesion	6,25	2,82	T
CD34	Cell adhesion	7,79	2,85	T
PAX6^d^	Transcription factor	2,32	8,64	T
NOG	TGFβ signaling pathway	8,18	5,76	T, Q
CDX2^d^	Transcription factor	2,60	12,80	Q
COL2A1	Extracellular matrix	20,51	227,51	T

a NCBI Entrez Gene Information.

b T: ABI TaqMan Stem Cell Pluripotency Array; Q: qPCR assay, primers listed in supplementary Text S1.

c Promoter bound by NANOG but not OCT4/SOX2 in hESC [3].

d Promoter bound by NANOG and OCT4/SOX2 in hESCs [3].

Of those genes expressed in NANeG cells (Tables 2 and S1), 45% were differentially expressed (up- or downregulated ≥2-fold in both cell lines) between NANOG^high^ and NANOG^low^ cells (Table 2). Twenty-one genes were upregulated in NANOG^high^ cells, most of which were classified as stem cell markers [35]. Interestingly, the mesoderm/primitive streak markers *T, EOMES* and *MIXl1* were also upregulated in NANOG^high^ cells compared to NANOG^low^ cells. Amongst the 11 genes commonly downregulated in NANOG^high^ cells were the extracellular matrix-encoding genes *COL1A1*, *COL2A1*, *FN1* and *LAMA1*, as well as *CDX2*, *ACTC1* and *PAX6*, which mark trophoblast, cardiac and neural differentiation, respectively. Several genes regulating cell adhesion were differentially expression in the sorted populations: CDH1/E-cadherin, PODXL and CD9 were upregulated in NANOG^high^ cells whereas CDH5/VE-cadherin and CD34 were downregulated in NANOG^high^ cells. CDH2/N-cadherin was not differentially expressed between NANOG^high^ and NANOG^low^ cells. Finally, components of the transforming growth factor beta signaling pathway were amongst those genes with the strongest difference in expression levels between NANOG^high^ and NANOG^low^ cells. Thus, *NODAL*, *LEFTY1*, *TDGF1* and *GDF3* were strongly upregulated in NANOG^high^ cells while *NOGGIN* showed a prominent downregulation in NANOG^high^ cells.

## Discussion

NANOG is an essential mediator of pluripotency in mammalian embryos and in cultured pluripotent stem cells [6], [7], [9] and is required to induce full pluripotency during reprogramming or cell fusion experiments [10], [11]. This makes NANOG an interesting candidate gene to study the biological mechanism of pluripotency *in vitro*. The detection of NANOG expression in hESCs has depended on methods which require fixation and processing of cells, which is labor-intensive and prevents the detection of NANOG expression in real-time in viable cultures. To address this, we describe here gene targeting of the *NANOG* locus in hESCs to obtain fluorescent NANOG reporter cell lines. *NANOG* gene targeting was performed with a BAC-derived targeting vector and yielded comparable efficiencies to previous reports on gene targeting in hESCs [36], [37], [38], [39], [40]. NANeG reporter cell lines maintained pluripotency as shown by hESC-specific marker expression and multilineage differentiation in embryoid bodies. The eGFP reporter gene showed a graded expression pattern in undifferentiated cultures and was low or absent in a subset of hESCs, as previously reported for mESC Nanog reporter lines [9], [20]. Co-expression of NANOG and eGFP protein was detected in hESCs in undifferentiated cultures, but was coordinately lost in areas of spontaneous differentiation. However, a subset of hESCs stained positive for NANOG but showed no expression of eGFP. This discrepancy was also observed in mESC lines targeted at the Nanog locus [20] and could be due to different stability of NANOG and eGFP mRNA and/or protein. Alternatively, the eGFP-reporter containing allele may be selectively silenced in NANeG cells by yet unidentified mechanisms. Reporter gene expression was responsive to cell culture conditions which induce or repress *NANOG* expression *in vitro*. Thus, *NANOG* and eGFP were concomitantly downregulated during hESC differentiation, whereas the addition of Activin A, which directly activates the *NANOG* promoter in hESCs via its downstream effectors SMAD2 and SMAD3 [6], [8], increased reporter gene expression.

Embryonic stem cells are a heterogeneous cell population, consisting of subpopulations with variant expression levels of pluripotency-associated markers and differentiation status [41]. Thus, murine ESCs show a heterogenous pattern of Nanog expression, and Nanog^high^ and Nanog^low^ subpopulations are characterized by differential gene expression [19]. Similar to mESCs, hESCs show a heterogeneous expression pattern of NANOG in undifferentiated cells [42]. The generation of fluorescent NANOG reporter lines facilitated the isolation and characterization of hESC subpopulations with distinct NANOG expression levels. Gene expression analysis of 96 genes involved in stem cell pluripotency or differentiation was carried out to identify distinct gene expression patterns in NANOG^high^ and NANOG^low^ subpopulations. Expectedly, we detected higher expression levels of genes associated with hESC pluripotency (including NANOG itself) in NANOG^high^ versus NANOG^low^ hESCs. Conversely, differentiation markers for embryonic and extraembryonic tissues (*ACTC1*, *PAX6*, *CDH5*, *CDX2*, *CD34*) and extracellular matrix proteins (*COL2A1*, *COL1A1*, *LAMA1*, *FN1*) were upregulated in NANOG^low^ hESCs. A similar upregulation of extracellular matrix genes has been found in Nanog^low^ cell isolated from mESCs [19]. The primitive endoderm markers GATA4 and GATA6, which were upregulated in Nanog^low^ mESCs [19] were upregulated in NANOG^low^ cells of NANeG1 but slightly downregulated in NANOG^low^ cells of NANeG3. Interestingly, genes involved in primitive streak formation and mesoderm differentiation (*NODAL*, *T*, *EOMES*, *MIXL1*) were upregulated in the NANOG^high^ fraction. This observation is consistent with high Nanog expression levels in the area of primitive streak formation in the mouse embryo, where Nanog expression co-localized with the primitive streak marker Mixl1 [42], [43].

Promoter binding of NANOG has previously been studied on 18.000 annotated genes in hESCs [3]. Thereby, it was found that NANOG binds to over 1600 promoters of both active and inactive genes in hESCs, and that the majority of promoters bound by NANOG were co-occupied by OCT4 and SOX2. When comparing the list of genes differentially expressed in NANOG^high^ and NANOG^low^ cells with the published promoter binding data, we found that 14 out of 32 gene promoters (44%) were bound by NANOG, indicating that they are direct transcriptional targets of NANOG. Moreover, five genes differentially expressed in NANOG^high^ and NANOG^low^ cells (*NODAL*, *T*, *GDF3*, *PODXL* and *ZFP42*) were bound by NANOG but not OCT4 and SOX2, indicating that NANOG plays a unique role in regulating expression of these genes. In contrast, of those genes not differentially expressed between NANOG^high^ and NANOG^low^ cells, 12 out of 39 (31%) were co-occupied by NANOG, OCT4 and/or SOX2, but none was bound by NANOG only.

Previous knockdown studies performed to study the role of NANOG in hESCs yielded variable results with respect to changes in downstream gene expression, probably reflecting differences in culture system and experimental design between those studies [4], [5], [6], [44]. In contrast, NANeG lines provide a novel system to study spontaneous fluctuations in NANOG expression levels within hESC cultures and concomitant changes in gene expression patterns, which may lead to the identification of NANOG target genes under steady-state hESC culture conditions.

In conclusion, we successfully derived fluorescent NANOG reporter lines by gene targeting in hESCs. Reporter gene expression responded to stimuli that modulate NANOG expression with appropriate changes in expression levels. Future applications of NANeG lines include the identification of novel regulators of NANOG expression and hESC pluripotency (e.g. in high throughput screens with chemical, cDNA or shRNA libraries) and the separation and extensive characterization of hESC subpopulations with distinct NANOG expression levels to clarify the role of NANOG in hESC pluripotency and differentiation.

## Supporting Information

Figure S1 Expression of markers for pluripotency and differentiation in NANeG1. A) Undifferentiated NANeG1 cells growing on murine feeders were stained for OCT4, SSEA-4, TRA-1-60 and TRA-1-81 as indicated. Scale bar: 100µm. B) NANeG1 cells were differentiated to embryoid bodies and subsequently plated on matrigel-coated culture dishes. Differentiation to the three embryonic germ layers was assessed by staining for beta-3-tublin (TUBB3, ectoderm), alpha-fetoprotein (AFP, endoderm), albumin (ALB, endoderm) and alpha-smooth-muscle actin (alpha-SMA, mesoderm). Scale bar: 100µm. C) Semi-quantitative reverse transcriptase polymerase chain reaction was used to detect expression of germ-layer-specific markers in NANeG1 cells differentiated to embryoid bodies. Samples were taken at the indicated timepoints (day 0 to day 18) and tested for expression of the ectodermal markers SOX1 and neurofilament (NFM), the mesodermal markers T and CD31, the endodermal markers alpha-fetoprotein (AFP) and albumin (ALB) and the trophectodermal marker CDX2. Amplification of glyceraldehyde 3-phosphate dehydrogenase (GAPDH) cDNA was used as internal control.
(2.81 MB TIF)

Figure S2 Expression of markers for pluripotency and differentiation in NANeG3. A) Undifferentiated NANeG3 cells growing on murine feeders were stained for OCT4, SSEA-4, TRA-1-60 and TRA-1-81 as indicated. Scale bar: 100µm. B) NANeG3 cells were differentiated to embryoid bodies and subsequently plated on matrigel-coated culture dishes. Differentiation to the three embryonic germ layers was assessed by staining for beta-3-tublin (TUBB3, ectoderm), SOX1 (ectoderm), alpha-fetoprotein (AFP, endoderm) and alpha-smooth-muscle actin (alpha-SMA, mesoderm). Scale bar: 100µm. C) Semi-quantitative reverse transcriptase polymerase chain reaction was used to detect expression of germ-layer-specific markers in NANeG3 cells differentiated to embryoid bodies. Samples were taken at the indicated timepoints (day 0 to day 28) and tested for expression of the ectodermal markers SOX1 and PAX6, the mesodermal markers T, cardiac troponin T (cTNT) and CD31, the endodermal markers alpha-fetoprotein (AFP), SOX17 and albumin (ALB) and the trophectodermal marker CDX2. Amplification of beta-actin (ACTB) cDNA was used as internal control.
(3.22 MB TIF)

Figure S3 Copy number of integrated targeting vector in NANeG cells. A) Relative quantities of the NANOG proximal promoter (pNANOG) and the single-copy genes GDF3 and FOXJ2 were measured on a dilution series of genomic DNA from wildtype hESCs. Threshold cycle (Ct) values from three independent experiments were plotted against log-transformed concentrations of genomic DNA. Trend lines were inserted and used to obtain values for slope (y) and correlation coefficients (R2). B) Quantities of pNANOG relative to GDF3 (upper panel) and FOXJ2 (lower panel) were determined for three wildtype hESC lines (HUES-3, HUES-1 and H9, grey bars), four NANeG clones (clones 1, 3, 31 and 11, white bars) and three clones with random transgenic insertion of the NANOG targeting vector (clones 12, 13 and 32, black bars). Plots show average and standard deviations obtained from at least three independent experiments. The mean of pNANOG vs.GDF3 and pNANOG vs. FOXJ2 in the three wildtype hESC lines is included in both figures (striped bar) and was used for statistical analyses to identify samples with significantly increased amounts of pNANOG.
(0.06 MB PDF)

Figure S4 Isolation of eGFPhigh and eGFPlow populations from NANeG cells. A) Flow cytometry profiles of NANeG1 and NANeG3 during cell sorting. Gates for eGFPhigh and eGFPlow cells are indicated. B) Expression of eGFP in eGFPlow cells relative to eGFPhigh cells isolated from NANeG lines was measured by quantitative PCR.
(0.06 MB PDF)

Text S1 (0.08 MB DOC)

Table S1 Genes not differentially expressed (less than 2-fold up/downregulated in one or both cell lines) in NANOGhigh and NANOGlow hESCs.
(0.02 MB XLS)
