# Supplementary figures and images for: NANOG Reporter Cell Lines Generated by Gene Targeting in Human Embryonic Stem Cells
Source: PLoS One. 2010 Sep 2;5(9):e12533. doi: 10.1371/journal.pone.0012533 (PMC2932718; doi:10.1371/journal.pone.0012533)

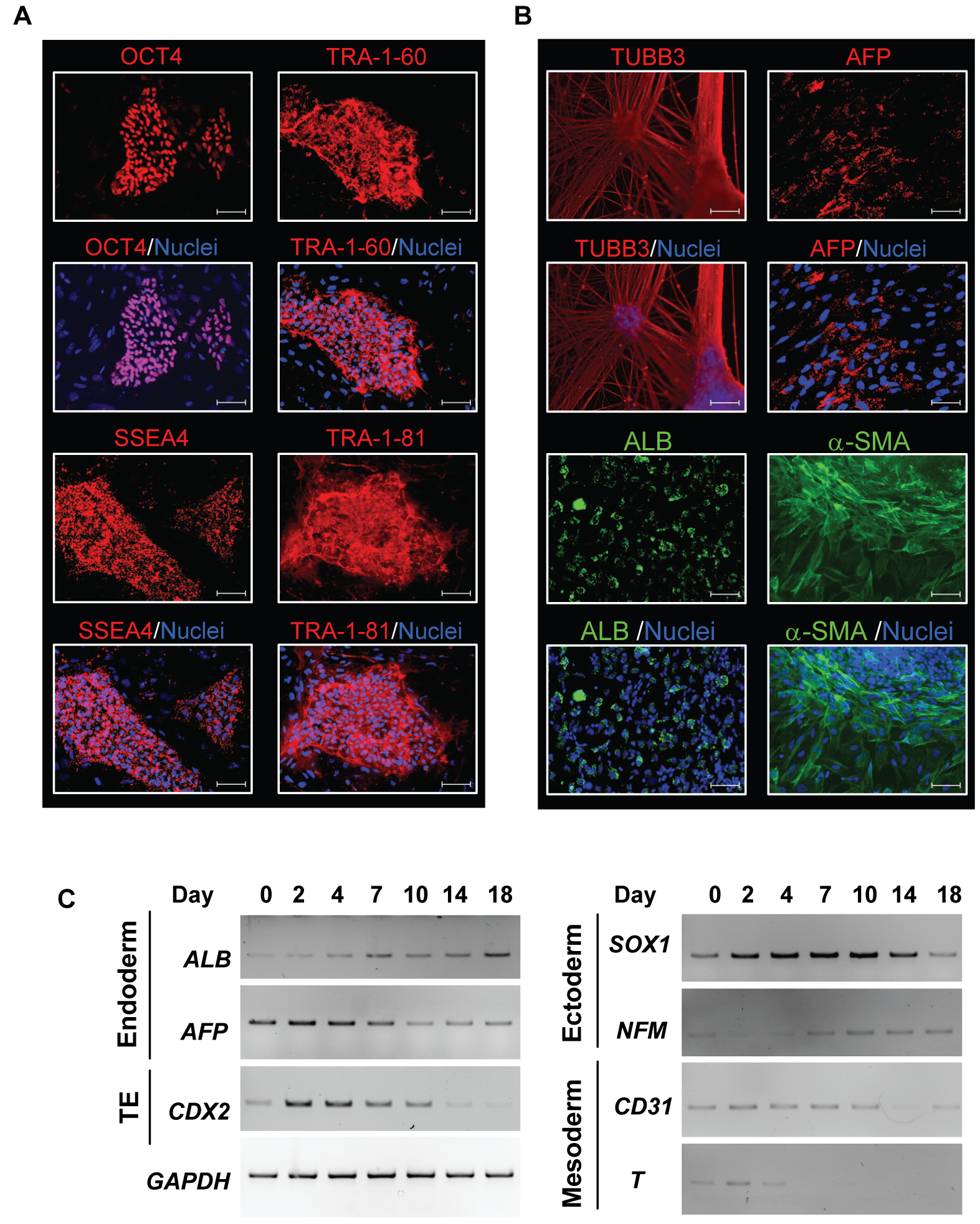

Supplement: Figure S1 — Expression of markers for pluripotency and differentiation in NANeG1. A) Undifferentiated NANeG1 cells growing on murine feeders were stained for OCT4, SSEA-4, TRA-1-60 and TRA-1-81 as indicated. Scale bar: 100µm. B) NANeG1 cells were differentiated to embryoid bodies and subsequently plated on matrigel-coated culture dishes. Differentiation to the three embryonic germ layers was assessed by staining for beta-3-tublin (TUBB3, ectoderm), alpha-fetoprotein (AFP, endoderm), albumin (ALB, endoderm) and alpha-smooth-muscle actin (alpha-SMA, mesoderm). Scale bar: 100µm. C) Semi-quantitative reverse transcriptase polymerase chain reaction was used to detect expression of germ-layer-specific markers in NANeG1 cells differentiated to embryoid bodies. Samples were taken at the indicated timepoints (day 0 to day 18) and tested for expression of the ectodermal markers SOX1 and neurofilament (NFM), the mesodermal markers T and CD31, the endodermal markers alpha-fetoprotein (AFP) and albumin (ALB) and the trophectodermal marker CDX2. Amplification of glyceraldehyde 3-phosphate dehydrogenase (GAPDH) cDNA was used as internal control. (2.81 MB TIF) [file pone.0012533.s001.tif]

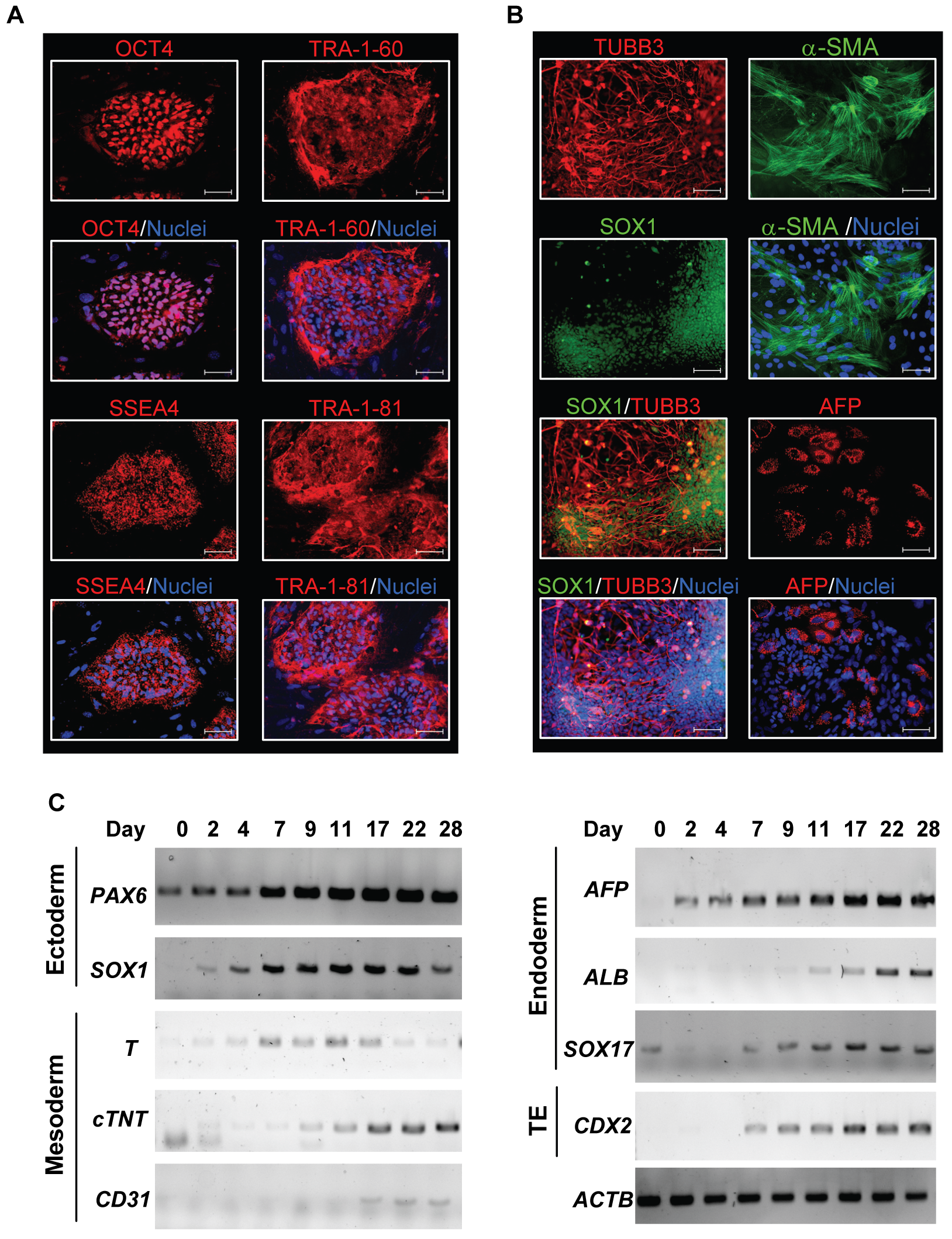

Supplement: Figure S2 — Expression of markers for pluripotency and differentiation in NANeG3. A) Undifferentiated NANeG3 cells growing on murine feeders were stained for OCT4, SSEA-4, TRA-1-60 and TRA-1-81 as indicated. Scale bar: 100µm. B) NANeG3 cells were differentiated to embryoid bodies and subsequently plated on matrigel-coated culture dishes. Differentiation to the three embryonic germ layers was assessed by staining for beta-3-tublin (TUBB3, ectoderm), SOX1 (ectoderm), alpha-fetoprotein (AFP, endoderm) and alpha-smooth-muscle actin (alpha-SMA, mesoderm). Scale bar: 100µm. C) Semi-quantitative reverse transcriptase polymerase chain reaction was used to detect expression of germ-layer-specific markers in NANeG3 cells differentiated to embryoid bodies. Samples were taken at the indicated timepoints (day 0 to day 28) and tested for expression of the ectodermal markers SOX1 and PAX6, the mesodermal markers T, cardiac troponin T (cTNT) and CD31, the endodermal markers alpha-fetoprotein (AFP), SOX17 and albumin (ALB) and the trophectodermal marker CDX2. Amplification of beta-actin (ACTB) cDNA was used as internal control. (3.22 MB TIF) [file pone.0012533.s002.tif]

**A**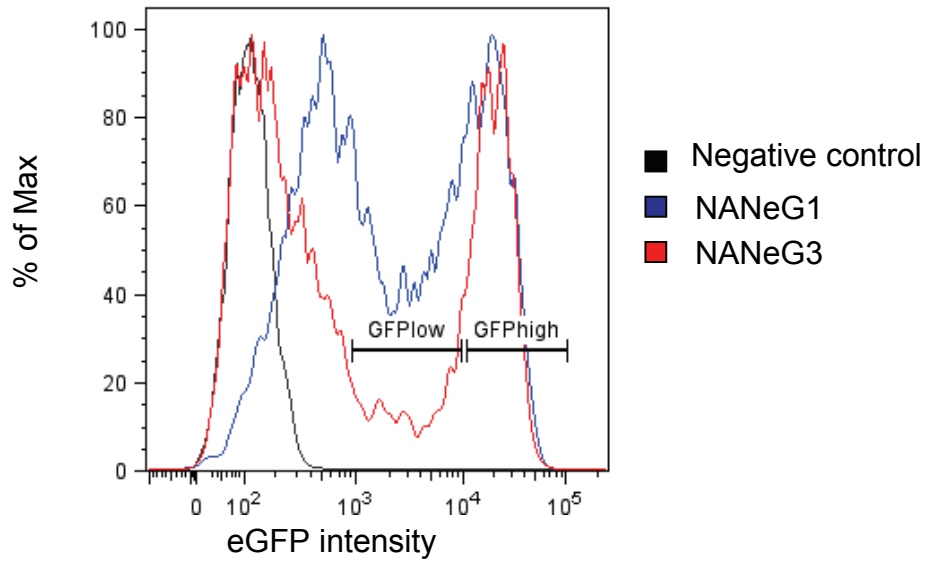**B**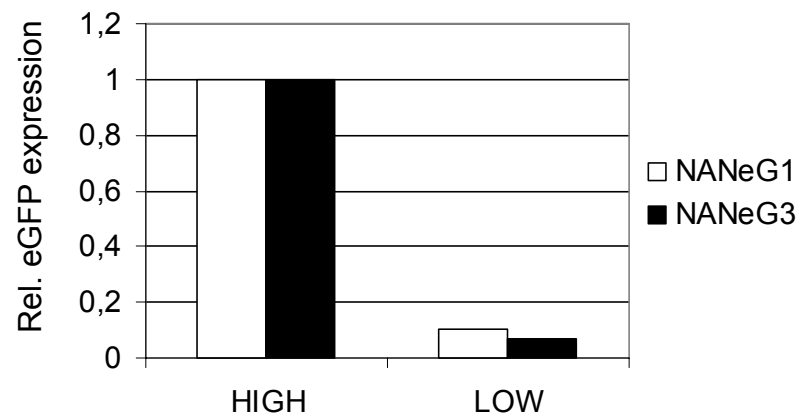

Supplement: Figure S4 — Isolation of eGFPhigh and eGFPlow populations from NANeG cells. A) Flow cytometry profiles of NANeG1 and NANeG3 during cell sorting. Gates for eGFPhigh and eGFPlow cells are indicated. B) Expression of eGFP in eGFPlow cells relative to eGFPhigh cells isolated from NANeG lines was measured by quantitative PCR. (0.06 MB PDF) [file pone.0012533.s004.pdf]
