# Supplementary material for: NANOG Reporter Cell Lines Generated by Gene Targeting in Human Embryonic Stem Cells
Source: PLoS One. 2010 Sep 2;5(9):e12533. doi: 10.1371/journal.pone.0012533 (PMC2932718; doi:10.1371/journal.pone.0012533)
Supplement: Figure S3 — Copy number of integrated targeting vector in NANeG cells. A) Relative quantities of the NANOG proximal promoter (pNANOG) and the single-copy genes GDF3 and FOXJ2 were measured on a dilution series of genomic DNA from wildtype hESCs. Threshold cycle (Ct) values from three independent experiments were plotted against log-transformed concentrations of genomic DNA. Trend lines were inserted and used to obtain values for slope (y) and correlation coefficients (R2). B) Quantities of pNANOG relative to GDF3 (upper panel) and FOXJ2 (lower panel) were determined for three wildtype hESC lines (HUES-3, HUES-1 and H9, grey bars), four NANeG clones (clones 1, 3, 31 and 11, white bars) and three clones with random transgenic insertion of the NANOG targeting vector (clones 12, 13 and 32, black bars). Plots show average and standard deviations obtained from at least three independent experiments. The mean of pNANOG vs.GDF3 and pNANOG vs. FOXJ2 in the three wildtype hESC lines is included in both figures (striped bar) and was used for statistical analyses to identify samples with significantly increased amounts of pNANOG. (0.06 MB PDF) [file pone.0012533.s003.pdf]

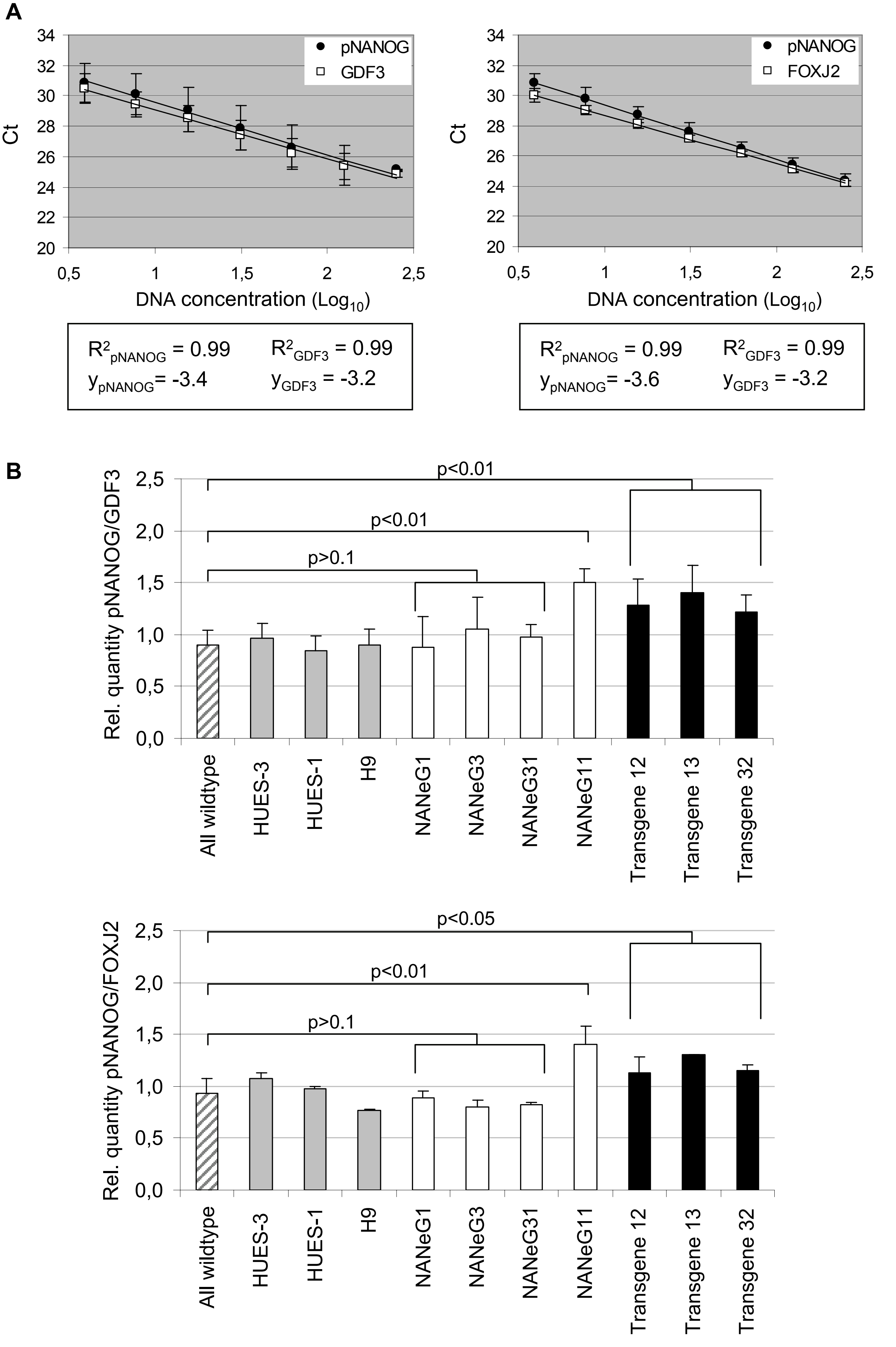
A
pNANOG
GDF3
Ct
DNA concentration (Log10)
R2pNANOG = 0.99
R2GDF3 = 0.99
ypNANOG= -3.4
yGDF3 = -3.2
pNANOG
FOXJ2
Ct
DNA concentration (Log10)
R2pNANOG = 0.99
R2GDF3 = 0.99
ypNANOG= -3.6
yGDF3 = -3.2
B
Rel. quantity pNANOG/GDF3
p<0.01
p<0.01
p>0.1
All wildtype
HUES-3
HUES-1
H9
NANeG1
NANeG3
NANeG31
NANeG11
Transgene 12
Transgene 13
Transgene 32
Rel. quantity pNANOG/FOXJ2
p<0.05
p<0.01
p>0.1
All wildtype
HUES-3
HUES-1
H9
NANeG1
NANeG3
NANeG31
NANeG11
Transgene 12
Transgene 13
Transgene 32
