# Supplementary material for: NANOG Reporter Cell Lines Generated by Gene Targeting in Human Embryonic Stem Cells
Source: PLoS One. 2010 Sep 2;5(9):e12533. doi: 10.1371/journal.pone.0012533 (PMC2932718; doi:10.1371/journal.pone.0012533)
Supplement: Text S1 — (0.08 MB DOC) [file pone.0012533.s005.doc]

**Primers for creating the NANOG targeting vector**

| **Reaction** | **Sequence Forward Primer** | **Sequence Reverse Primer** |
| --- | --- | --- |
| pSV40-Neo^R^ amplification^a^ | TCACTACGTGATAACTTCGTATAATGTATGCTATACGAAGTTATaaccatcaccctcctcaagttt | TGGTCTCGGTGGGGTATAACTTCGTATAGCATACATTATACGAAGTTATatcgacagagygccagccct |
| Reporter cassette insertion into BAC^b^ | ACCTTTTTTCCAGTCCACCTCTTAAATTTTTTCCTCCTCTTCCTCTATACTAACagcgctaccggactcagatc | GGATGCTTCAAAGCAAGGCAAGCTTTGGGGACAAGCTGGATCCACACTCATatatatgagtaacctgaggc |
| Retrieval^b^ | AGCTGTGTGTACTCAATGATAGATTTCAGAGACAGAAATACCTCAGCCTCTAGGGATAACAGGGTAATgaatgaatcaccgatacgcga | AGTTGCTAAAATTTACCAAGATGTCCTTCAGTAGGGTGAATGGATAAACTACCGGTttcttagacgtcaggtccac |

^a^ LoxP sites are capitalized, restrictions sites for DraIII (forward primer) and BsaI (reverse primer) are underlined.

^b^ 50bp of homology to the BAC target region are capitalized. The I-SceI site is underlined.

**Primers for detection of *NANOG*** gene targeting

| **Primer alias** | **Sequence** |
| --- | --- |
| Neo/F | CGCACGGTGTTGGGTCGT |
| Ex3/R | CTGGAACCAGGTCTTCACCTG |
| 5´/F | AAGCCCAGGCGCTAGTCTATAAGT |
| bGL/R | GAAAGGCAGGATGATGACCAGGAT |

**Primers for semi-quantitative PCR**

| **Assay** | **Forward primer** | **Reverse primer** | **Tm** | **Cycles** |
| --- | --- | --- | --- | --- |
| *ActinB* | CTCTTCCAGCCTTCCTTCCT | TGTTGGCGTACAGGTCTTTG | 56 | 35 |
| *AFP* | GCGAGGGAGCGGCTGACATT | GCGCTACACCCTGAGCTTGGC | 53 | 35 |
| *ALB* | GCAAGGCTGACGATAAGGAG | TGGCTTTACACCAACGAAAA | 53 | 35 |
| *CD31* | CCTGTCTTTCAGCCTTCAGC | CGCCTGTGAAATACCAACCT | 55 | 35 |
| *CDX2* | AGAGGGACTCAAGGGAAAGG | GGTCTGGGAAGGGAAGAGAA | 55 | 35 |
| *cTNT* | ATGATGCATTTTGGGGGTTA | CAGCACCTTCCTCCTCTCAG | 51 | 35 |
| *GAPDH* | GTTCGACAGTCAGCCGCATC | GGAATTTGCCATGGGTGGA | 55 | 30 |
| *NFM* | GAGGAACACCAAGTGGGAGA | TTCTGGAAGCGAGAAAGGAA | 56 | 35 |
| *PAX6* | GAACAGACACAGCCCTCACA | ATCATAACTCCGCCCATTCA | 53 | 35 |
| *SOX1* | cacaactcggagatcagcaa | ggtacttgtaatccgggtgc | 55 | 35 |
| *SOC17* | AAGGGCGAGTCCCGTATC | TTGTAGTTGGGGTGGTCCTG | 55 | 35 |
| *T* | Gatcacttctttcctttgcatcaag | tgcttccctgagacccagtt | 55 | 35 |

**Primers for quantitative** real-time PCR

| **Assay** | **Forward primer** | **Reverse primer** |
| --- | --- | --- |
| *NANOG* | GGTGGCAGAAAAACAACTGG | CATCCCTGGTGGTAGGAAGA |
| *GAPDH* | GTTCGACAGTCAGCCGCATC | GGAATTTGCCATGGGTGGA |
| *eGFP* | ATGGTGAGCAAGGGCGAGG | AGCACTGCACGCCGTAGGT |
| *NODAL* | GAGTTTCATCCGACCAACCA | ATCAGAGGCACCCACATTCT |
| *CDH1* | CTGCCATTCTGGGGATTCT | CTCTTCTCCGCCTCCTTCT |
| *TDGF* | CTACGACCTTCTGGGGAAAA | AGAAATGGCCATGATCCAAA |
| *POU5F1* | CGAAAGAGAAAGCGAACCAG | AACCACACTCGGACCACATC |
| *T ^1^* | Gatcacttctttcctttgcatcaag | tgcttccctgagacccagtt |
| *GDF3* | TACTTCGCTTTCTCCCAGACC | GCCAATGTCAACTGTTCCCT |
| *EOMES* | TCTGTGGCTCAAATTCCACC | TTTTGTTGCCCTGCATGTTA |
| *MIXL1* | CACCCTGCTCCCCGAGTCCA | AGATGCCCCCTCCAACCCCG |
| *ZFP42 ^2^* | TCACAGTCCAGCAGGTGTTTG | TCTTGTCTTTGCCCGTTTCT |
| *NOG* | GCCAGCACTATCTCCACATC | ATCCTTTTCCTTGGGGTCAA |
| *CDX2* | AGAGGGACTCAAGGGAAAGG | GGTCTGGGAAGGGAAGAGAA |
| *AFP* | CTTTGGGCTGC CG TATGA | TGGCTTGGAAAGTTCGGGTC |
| *CDH2* | CGACGAATGGATGAAAGACC | CAAGGACCCAGCAGTGGA |
| *CER1* | CTTCTCAGGGGGTCATCTTG | TCCCAAAGCAAAGGTTGTTC |
| *GATA6* | CGGCTTGGATTGTCCTGT | GGTTTTCGTTTCCTGGTTTG |
| *SNAIL* | GCACATCCGAAGCCACAC | GGAGAAGGTCCGAGCACAC |
| *SOX2* | ACACCAATCCCATCCACACT | cctccccaggttttctctgt |
| *SOX7* | CACTGCTGAACTGGTCCCTAA | TGGCTGGTGAGGAAGACAA |
| *eGFP* | ATGGTGAGCAAGGGCGAGG | AGCACTGCACGCCGTAGGT |

**Primers for *NANOG* promoter copy number determination**

| **Assay** | **Forward primer** | **Reverse primer** |
| --- | --- | --- |
| *pNANOG* | GGATAGACAAGAAACCAAACTAAAGGA | ATGGCATTATAGCATGAAGCCA |
| *GDF3* | CCTGGTTCAGGAGCCTCATG | TGAAGTGAACAGCACCTTGTGG |
| *FOXJ2* | TCTACGACAGGCAGAGCAGAAG | TGACCAGTCTGAGTAAGGAAGTGGT |

**1.** Osafune K, Caron L, Borowiak M, et al. Marked differences in differentiation propensity among human embryonic stem cell lines. Nat Biotechnol*.* Mar 2008;26(3):313-315.

**2.** Darr H, Mayshar Y, Benvenisty N. Overexpression of NANOG in human ES cells enables feeder-free growth while inducing primitive ectoderm features. Development*.* Mar 2006;133(6):1193-1201.
